# The Tortoise and the Hare: Guinea Worm, Polio and the Race to Eradication

**DOI:** 10.1371/currents.outbreaks.16e2349da74ec9bdfe26cc6598bee881

**Published:** 2015-08-31

**Authors:** Brett Sutton, Deon Canyon

**Affiliations:** Department of Health and Human Services, Melbourne, Victoria, Australia; Office of Public Health Studies, University of Hawaii at Manoa, Honolulu, Hawaii, USA

**Keywords:** afghanistan, eradication, Nigeria, pakistan, polio, south sudan

## Abstract

Introduction: The eradication of a human infectious disease is a major challenge and, if achieved, represents a enormous achievement. This article explores the long and difficult journey towards eradication for polio and guinea worm.

Methods: The authors reviewed the programmatic approaches taken in the eradication strategies for these two diseases and the unique socio-political contexts in which these strategies are couched. The epidemiology of the last 15 years is compared and contrasted. The specific challenges for both programs are outlined and some key elements for success are highlighted.

Discussion: The success of these eradication programs is contingent upon many factors. Nothing is assured, and progress remains fragile and vulnerable to setbacks. Security must be ensured in guinea worm transmission areas in Africa and polio transmission areas in Pakistan and Afghanistan. Technical solutions alone cannot guarantee eradication. National leadership and continued international focus and support are necessary, today more than ever. The legacy of success would be extraordinary. It would reverberate to future generations in the same way that the eradication of smallpox does for this generation.

## Introduction

Smallpox is no longer with us. Rinderpest, a measles-like virus of cattle, was formally declared extinct in 2011.^1^ What other diseases might follow? This paper looks at some clear candidates that were due for eradication this year and explores the chances of success and remaining obstacles for guinea worm and polio. The race is on.

The classic fable of Æsop is known almost universally by both children and adults. Somehow the tale of an overconfident hare losing a race to a much maligned and slower tortoise has entered our consciousness, although the exact moral lesson is ambiguous. Should we accept the conventional wisdom that ‘slow and steady wins the race’ or is the salient lesson one of not taking on a challenge when filled with hubris; or is it a cautionary tale about having callous over-confidence? Whatever the intention in the fable, there is perhaps a message contained therein that is pertinent to another race – the race to disease eradication. Not since the last case of smallpox in the wild was seen in Somalia in 1977 has a human scourge disappeared from the face of the earth.^2^ Yet now we are perhaps on the edge of seeing the next plague head to extinction. In 2015, polio and guinea worm can rightly be given the monikers of the hare and tortoise. And the race is not over yet.

Guinea worm, or more properly *Dracunculus medinensis* (‘little dragon from Medina’), is a member of the nematode family that may well be the ‘fiery serpent’ described in the Old Testament. Certainly it inflicts a terrible burning pain to the sufferer as the worm emerges. Remnants have been found in ancient Egyptian mummies.^3^ The Ebers papyrus, the famous Egyptian medical text, described a method of extraction that is still in use today.^4^ And the mode of infection reads like a horror story. After drinking water containing the nematode larvae carried within copepods - a small freshwater crustacean - the sufferer will be blissfully unaware as the female worm goes on to mate with a male worm in a body cavity. The male worm then dies and the fertilized female worm migrates into subcutaneous tissues towards the surface and grows up to 120 centimeters long and as thick as spaghetti. After perhaps a year, it then emerges with excruciating pain through a blister, often on lower limbs, but potentially from almost any bodily site, to deposit larvae on contact with water.^5^ It is this life cycle that was the first scientifically documented proof of a link between a vector and a disease, made by Alexei Pavlovich Fedchenko in 1870.^6^ Over a century later it was the pioneering work of Dr. Donald R. Hopkins that set guinea worm on the path to eradication. Then in 1988, ex-President Carter joined the commitment to eradicate this disease, prompted by witnessing a fist-sized abscess in the breast of a Ghanaian woman with a worm at its center.^7^

Polio has been with us for millennia, and is coincidentally also depicted in Egyptian history. Hieroglyphic steles depict a figure with a withered leg, almost certainly representing the classical manifestation of spinal polio, where a limb becomes paralyzed and later deformed. As for guinea worm, the race to eradicate polio began in 1988 under the Global Polio Eradication Initiative, which brought together a partnership between the World Health Organization (WHO), Rotary International, Centers for Disease Control and Prevention (CDC) and UNICEF.^8^ At the time, the feasibility of polio eradication was seen in the same light as smallpox had been seen. Eradication criteria were similar in that a vaccine was available and effective; the disease only existed in the human host, with no animal reservoir, and symptoms allowed for surveillance and identification of final cases.

## Approaches to eradication

Undeniably both of these programs have made remarkable progress since those first commitments to eradication were made (Fig. 1). Both have reduced endemicity to only a few countries and decreased the global burden of disease by over 99%. The approaches to eradication, however, could hardly be more different. Guinea worm is being defeated without a vaccine, without treatment being available, and behavioral change is central to the effort. Guinea worm’s life cycle is interrupted through simple physical barriers and human behaviors play a key role in their maintenance. For polio, vaccination is the alpha and omega of eradication.

**Cases of guinea worm (left Y axis — ) polio (right Y axis ---) from 2001 to 2014.**
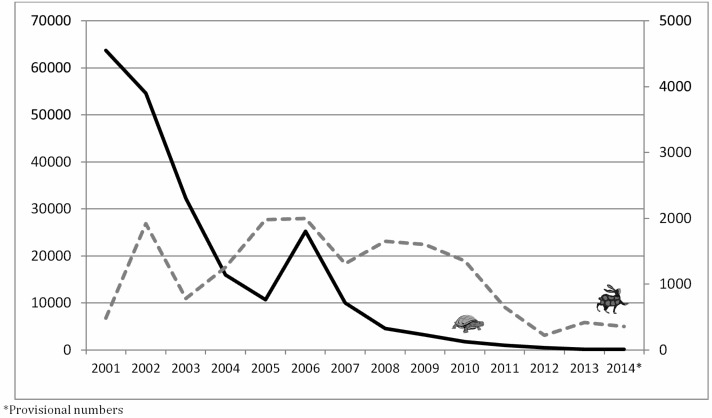
9 ^,^ 10

Guinea worm’s eradication program has focused on the provision of clean water; pipe and cloth filters to prevent ingestion of the worm; larviciding potentially infected water sources; and preventing the reintroduction of copepods into drinking water.^11^ This last action is key. As the guinea worm emerges from the skin and the burning pain becomes intolerable, the sufferer is inclined to soothe the affected limb in water, whereupon the female worm releases its larvae and re-infects resident copepods. The worm emerges incredibly slowly and extraction involves winding it around a stick a few millimeters a day until, some months later, it is removed intact. During this time patients are housed and wounds are dressed until they no longer pose a risk. The motto for years in the Carter Center and CDC’s ‘Guinea Worm Wrap-up’ was: Detect Every Case, Contain Every Worm!^12^

Like guinea worm, polio is untreatable, debilitating and disabling. A vaccine is, however, available - and essential - to prevent polio. This has been the cornerstone of the eradication effort from its inception. Once vaccination coverage is consistently high enough in areas where transmission is occurring, the virus ceases to circulate, and then – without an alternative host - ceases to exist. This strategy has seen polio disappear from the Americas, Europe, Oceania and the Western Pacific. Type 2 polio virus (WPV2) has already met with extinction. Type 3 (WPV3) has almost certainly followed with the most recent reported onset in a case in November 2012.^13^ If certified - following three years of surveillance with confirmed absence - that would leave type 1 as the last remaining wild polio type in existence.

So, is this a slow but steady movement towards certain victory? Will these public health investments be repaid a hundred-fold in decades to come? Perhaps, perhaps not. It is testament to the supreme efforts of the dedicated men and women in public health through the 1970s that smallpox eradication became a reality. It may also be a consequence of their success that the ambitious program to eradicate polio was conceived and committed to; a program that has missed deadlines and is as yet not fulfilled.

## Progress and challenges in polio eradication

At the beginning of 2002, the battle to eradicate polio looked near to won, at hare-like speed. The total number of cases globally in 2001 was 483 with only eight cases occurring in non-endemic countries. The number of endemic countries had fallen from 20 in 2001 down to 10 the following year. India had the majority of cases at a mere 268. Yet two years later in 2003 the global total had risen to over 1900 cases and India alone had 1600. Nigeria, which had 28 cases in the year 2000 had 1122 cases by 2006. As quickly as one country would stop transmission, another would become newly infected.^14^

What had gone wrong? This staggered progress illustrated a number of enormous challenges. India’s backward steps related to several technical, social and logistical challenges. Firstly, there was the challenge of delivering polio vaccines to poor, overcrowded regions, especially in the hugely populated northern states of Uttar Pradesh and Bihar, where even basic sanitation was often absent. These two states combined had over 500,000 births per month; all requiring vaccination before encountering the virus.^15^ Parts of Bihar spend almost six months submerged by India’s monsoonal rains, isolating literally thousands of villages and hamlets. Secondly, it was discovered that in these crowded and unsanitary conditions, virus transmission was the most highly efficient in the world.^16^ Finally, these northern states contained a small but significant percentage of families within a Muslim minority, suspicious of state interventions and concerned the polio vaccine was a means of population control.^17^

Nigeria demonstrated just how critical trust in the vaccine and those delivering it was to a successful eradication program. In late 2003, the northern states of Nigeria stopped polio vaccination altogether for several months, leading to a massive re-emergence of polio cases. Again the mistrust was in part based around a belief that polio vaccination was a state-sponsored mass fertility control measure. Only with the understanding and support of key religious figures in the north did vaccination recommence. Both northern Nigeria and northern India highlighted yet another substantial threat; that of the trans-boundary export of the virus to other countries. Viruses originating from Nigeria eventually spread to over twenty states across Africa and beyond to Yemen and Indonesia.^18^

These backward steps changed, however, with the development of what some public health officials called a ‘game-changer’. It was a bivalent oral polio vaccine for sub-types 1 and 3, more effective than the old trivalent and no worse than monovalent for inducing immunity to type 1. It has allowed sub-type 3 to be gradually squeezed out whilst making steady progress against type 1. India, with its enormous eradication challenges, defeated the last pocket of wild polio in January 2011. Nigeria, with unprecedented religious support from imams, had reduced its cases in 2014 to six, compared to 53 the previous year. In August 2015, the African continent will have gone one year without a single polio case for the first time in history. Indeed, its last case of polio may already have occurred and the hare’s confidence is perhaps justified.

Notwithstanding the financial burden of the final mile (now close to one billion USD^19^ per year), two other substantial obstacles remain; one technical and the other very human. The oral polio vaccine (OPV) has been used in the remaining endemic countries even as developed countries continue to use the inactivated polio vaccine (IPV), which is injected. The primary reason for this is that the oral form can continue to be shed from the gut of vaccine recipients, thereby contributing to ‘herd immunity’ and increasing *de facto* population immunity. IPV can’t do that. The downside is that OPV can mutate back to a form of the virus that clinically causes polio and then ‘circulate’ just like wild virus, causing polio in any vulnerable child. Cases continue to occur globally. Only when no cases of wild or vaccine-derived polio occur, all remaining OPV-using countries are expected to simultaneously switch to IPV, but the cost and logistics are both daunting.

The other, and potentially more difficult challenge, is ongoing conflict in the tribal areas of Pakistan. In 2010 Pakistan allowed an expanding outbreak of polio, and 2011 continued to see cases.^20^ The source of many of these is the administrative region of Federally Administered Tribal Areas (FATA) which saw a very active insurgency and extremely limited access to a large population of children. That changed in July 2014 when Pakistan’s army took control of previously inaccessible areas but this also saw a huge movement of people and the export of cases across the country. Progress has faltered. In 2014, the tenth report of the Independent Monitoring Board (IMB) on the Global Polio Eradication Initiative was scathing, labeling Pakistan’s polio program a “disaster” and highlighting the inertia in the response and the widespread pessimism regarding future prospects of eradication.^21^ The IMB’s entreaty for Pakistan to establish an Emergency Operations Center modeled after Nigeria’s highly successful center for polio and later Ebola has only just been heeded. Proof of effectiveness of the new national and provincial centers is eagerly awaited.

## Progress and challenges in guinea worm eradication

The tortoise, meanwhile, in the form of guinea worm’s eradication program, continues to plod along with barely a backwards step. In 2003, Uganda reported its last indigenous case, followed soon by Mauritania, Benin, Côte D’Ivoire, Burkina Faso and Togo in 2006. In February 2011, Niger and Nigeria were recognized for going 12 consecutive months without an indigenous case of guinea worm. For Nigeria it was all the more exceptional given that it was reporting 3.5 million cases annually when the eradication program started.

South Sudan remains the last real bastion for Dracunculiasis, with 1698 cases in 2010. Yet even here, reported cases have consistently dropped year by year.^22^ There were a mere 70 cases in 2014 and no cases for seven consecutive months to June 2015. This is South Sudan’s “peace dividend”, even as that peace is challenged. As development assistance arrives in a newly independent South Sudan, one can only hope that such progress (and assistance) will accelerate, and that a genuine peace will enable this particular dividend to be won.

Will the tortoise inevitably win this race? Or, as in Zeno’s paradox, will the finish line become ever more inaccessible even as it nears? Guinea worm certainly looks to be moving inexorably towards eradication. Yet in 2010, after a decade of no cases, a disturbing report of 10 cases in Chad occurred, following discovery by a WHO monitoring mission in July. More worryingly, the origin of these cases could not be determined. Every year since 2010 Chad has seen further human cases across a wider geographical area, re-initiating its endemic status. Now Dracunculiasis infections in dogs – first noted in 2012 – is presenting an even more worrying hypothesis. The worm specimens from dogs appear to be morphologically and or genetically confirmed as *D. medinensis* and indistinguishable from human specimens. It seems that human and canine cases are associated with Chad’s intense fishing industry. Human and canine cases appear to occur following consumption of undercooked or raw fish respectively.^23^ The way forward for prevention of cases and treatment of contaminated water sources is not entirely clear in this case.

## Reflections on the finish line

So the hare, in the polio eradication program, is definitely taking periodic naps. Any hubris that might have existed in the past has been surely tempered by the setbacks of recent years, the backwards steps of Pakistan’s program and the emerging complexity of what lies ahead. The hare may be racing in Africa, but he is currently enjoying his repose in Pakistan.

The tortoise, in the form of the guinea worm eradication program, can definitely see the finish line. President Carter says that eradication will happen in his lifetime – he is 90. The final cases may well be in Chad, with the very last worm emerging perhaps in 2016 or 2017. Is that time enough to win? Will a good understanding of Chad’s curious epidemiology arrive in time to facilitate this victory? A backwards step now would be uncharacteristic of this plodding tortoise. Only time will tell.

So, what lies in the future for the hare and the tortoise? These authors are placing their bets on guinea worm to become the third eradicated pathogen. Polio eradication, on the other hand, is sitting on a knife-edge. Now is almost certainly the last chance for polio eradication since donor funds will dry up if progress continues to sputter along. The conflict-ridden areas of Pakistan and Afghanistan may continue to facilitate virus circulation for some months, perhaps years. The intractable hotspot of North Waziristan in FATA remains a hotbed of polio production in Pakistan. Urgently accessing the thousands of children who have never received a vaccination there is a necessary part of the solution. Emergency Operation Centers armed with the clarity, purpose and energy evidenced in Nigeria’s EOC would be a good start. The ‘peace dividend’ for polio would be incalculable. And a fable to rival one of Æsop’s could well be written.

## Competing Interests

The authors have declared that no competing interests exist.
